# Partial replacement of soybean meal with different protein sources in piglet feed during the nursery phase

**DOI:** 10.5713/ajas.17.0753

**Published:** 2018-10-26

**Authors:** Jansller Luiz Genova, Paulo Levi de Oliveira Carvalho, Newton Tavares Escocard de Oliveira, Aparecida da Costa Oliveira, Franz Dias Gois, Davi Elias de Sá e Castro, Fábio Nicory Costa Souza, Heloíse Trautenmüller, Liliana Bury de Azevedo dos Santos, Isabela Ferreira Leal

**Affiliations:** 1 Animal Science Department, State University of Western Paraná, Marechal Cândido Rondon, PR 85960-000, Brazil; 2 Animal Science Department, State University of Santa Cruz, Ilhéus, BA 45662-900, Brazil; 3 Animal Science Department, Federal University of Bahia, Salvador, BA 40170-110, Brazil

**Keywords:** Nitrogen Balance, Growth Performance, Fish Meal, Blood Parameters, Soybean Protein Concentrate, Swine

## Abstract

**Objective:**

Evaluate the partial replacement of soybean meal with different protein sources in piglet feed during the nursery phase in terms of digestibility of feed, nitrogen balance, growth performance and blood parameters.

**Methods:**

Experiment I involved 24 crossbred entire male pigs with an initial body weight (BW) of 18.28±0.7 kg and used a randomized complete block design consisting of 3 treatments (fish meal, FM; soybean protein concentrate, SPC; and soybean meal, SBM) and 8 replicates, with 1 pig per experimental unit. Experiment II involved 1,843 crossbred male and female pigs with an initial BW of 6.79±0.90 kg and was based on a completely randomized design with a 2×3 factorial arrangement (2 sexes and 3 protein sources) and 13 replicates.

**Results:**

The results of Exp. I indicate effects (p<0.05) of dietary protein sources on digestible protein (FM, 17.84%; SPC, 16.72%, and SBM, 18.13%) and on total nitrogen excretion (TNE, g/kg BW^0.75^/d) in which pigs fed with SBM-based feed had TNE values that were 5.36% and 3.72% greater than SPC and FM, respectively. In the Exp. II, there was difference (p<0.01) between sexes in the pre-starter I and starter phases, and total period in average daily feed intake (ADFI), which were greater in females, and between the protein sources, ADFI, final weight and daily weight gain. For urea in the pre-starter II and starter phases and glucose in the pre-starter II phase, there was a difference (p<0.05) between protein sources and between sexes, in starter phase in urea concentrations (females: 57.11 mg/dL and males: 50.60 mg/dL).

**Conclusion:**

The use of SBM as only protein source influences larger TNE (g/kg BW^0.75^/d), reduces the growth performance of piglets and increases plasma urea concentrations in pre-starter II phase.

## INTRODUCTION

Weaning is considered the most critical phase in the life of a piglet because it is associated with numerous stress factors, such as loss of contact with the mother, environmental and dietary changes, and the establishment of a new social hierarchy. During this transitional period reduced feed intake, high morbidity and susceptibility to enteric infections are observed, considering that the development of the gastrointestinal tract and microbiota, and undeveloped active immunity are related to the growth performance of animals [1].

In this context, developments in animal nutrition provide information for the formulation of balanced diets, through better evaluation of ingredients and knowledge of the nutritional requirements of different phases of breeding. With the intensive production of pigs there is a need to search for new nutritional ingredients and strategies that contribute to the efficient functioning of the physiology of the animals and promote good growth performance.

It is important for animal nutrition to study ingredients that minimize production costs and, while at the same time promote health for piglets. The questions for nutritionists are the protein concentrations in nursery period, the sources of protein to formulate feeds for weaned piglets [2] and the digestibility and palatability of the feed. Therefore, soybean meal (SBM) is used in feeds as a primary source of vegetable protein. In contrast, piglet feeds are complex and, notably, make use of different protein sources that minimize the decreasing performance curve which occurs in the first days after weaning.

Studies evaluating the use of protein sources in the nutrition of piglets have only recently been reported and include experiments with soybean protein concentrate (SPC) and SBM [3,4], fish meal (FM) [5,6], spray-dried animal plasma and whole egg [7].

In this context, the goal of this study was to investigate the partial replacement of SBM with different sources of protein in piglet feed and their effect on feed digestibility, nitrogen balance, blood parameters and growth performance.

## MATERIALS AND METHODS

Two experiments were conducted. Experiment I was carried out in the Swine Sector of the Experimental Station Nucleus of the State University of Western Paraná - Unioeste, Campus of Marechal Cândido Rondon, Paraná. Experiment II was carried out in a commercial grange, located in the city of Marechal Cândido Rondon, PR.

### Animal care

All animal-based procedures and the protocol for animal use are in approval by the Animal Care and Use Committee of State University of Western Paraná - Unioeste.

### Experimental design, animals, housing and diets

A total of 24 crossbred entire male pigs, with an initial average body weight (BW) of 18.17±0.7 kg, were used in a metabolism study and were distributed in a randomized complete block design with 3 treatments (protein sources) and 8 replicates, totaling 24 experimental units. The blocks were based on initial BW.

The animals were housed individually in metabolic cages similar to those described previously [8], where they remained for 12 days, 7 days for acclimation to cages and feed, and 5 days for collection of feces and urine.

During the acclimation phase, the animals received two meals per day, provided at 08:00 and at 15:00. The total daily amount of feed provided during the collection period was determined from the acclimation period, based on the animals’ voluntary consumption and metabolic weight (BW^0.75^). The feed was moistened with water (20% by total weight of feed) to avoid waste, reduce powdery properties and improve palatability. After each meal, water was supplied through a feeder in the proportion of 3 mL/g of feed consumed; the quantity was calculated for each experimental unit to avoid excess water consumption.

A data logger, with a capacity of 24 measurements per d, was used to measure the ambient temperature (°C) and relative humidity during the experimental period. The minimum recorded temperature of the internal environment was 21°C ±4.95°C and the maximum was 23°C±5.16°C.

The experimental feeds were composed of: control diet containing soybean meal as a conventional protein source (SBM); control diet with the inclusion of fish meal (SBM+FM); and control diet with the inclusion of SPC (SBM+SPC; Table 1). The feed was provided by a local company and the guaranteed concentrations of nutrients were close to the reported concentrations [9]. The physicochemical composition values of all tested ingredients were determined by the NIRS DS2500 F analyzer (Nutrifarma, Maripá, PR, Brazil). The results are close to those found in the Brazilian Tables for Poultry and Swine: feed composition and nutritional requirements [9]. Four types of feed were provided for all piglet growth phases involved in the current study: post-weaning, pre-starter I, pre-starter II, and starter (Table 2).

For the performance trial (Exp. 2), 1,843 crossbreed piglets (998 entire males and 845 females) were used, aged 21 days with an average initial BW of 6.79±0.90 kg. Animals were distributed in a completely randomized design, in a 2×3 factorial arrangement (2 sexes and 3 protein sources) with 13 replicates of 46 to 53 animals per experimental unit. All treatments had the same number of replications, that is, experimental units.

The experimental diets were same used in Exp. 1. The animals were identified with numbered ear tags and housed in suspended nursery stalls (14 m^2^), with polyethylene plastic flooring, equipped with pacifier type drinking fountains and semi-automatic center feeders, and located in masonry shed with concrete flooring, ceramic roof tiles and a wood-furnace heating system. Feed and water were provided *ad libitum* throughout the experimental period.

A data logger, with a capacity of 24 measurements per day, was used to measure the ambient temperature (°C) and relative humidity during the experimental period. The minimum recorded temperature of the internal environment was 15°C± 5.73°C and the maximum was 16°C±5.78°C.

In Exp. 2, blood parameters were determined at the end of the pre-starter II and starter phases in 231 of the total animals tested. Blood samples were collected via the vena cava, using 0.70×30 mm and 0.80×40 mm gauge needles, into 5-mL test tubes containing urea anticoagulant (ethylenediaminetetraacetic acid) and glucose (sodium fluoride), according to the technique previously described [10]. Blood samples were then sent to the Blood Parameters Laboratory of the State University of Western Paraná. The samples were centrifuged (Centrilab digital centrifuge, Model 80-2B; Labingá, Maringá, PR, Brazil) at 3,000 g for 15 min; the plasma was transferred to “Eppendorf” polyethylene tubes in duplicate, labelled and stored in a freezer at −5°C for further analysis. Plasma urea and glucose concentrations were determined using kits from ELITech (Clinical Systems; ELITech Latin America, Vitória, ES, Brazil).

### Sample collection and preparation

In Exp. 1, the amount of feed supplied, feces and urine samples collection were conducted according to the methods previously described [11].

During the collection period, 1% of ferric oxide (Fe_2_O_3_) was added to the feed to mark the beginning and end of fecal collection. Feces were collected daily, weighed and placed in labelled plastic bags and stored in a freezer at −18°C. After the collection period, fecal samples were thawed, homogenized, weighed on a digital scale and a composite sample (20% by total weight of feces) of that collected from each experimental unit was removed. Composite samples were dried in a forced ventilation oven (55°C), ground in a Wiley type grinder mill and stored in polyethylene pots for analysis of dry matter (DM), organic matter (OM), mineral matter, gross energy (GE), and crude protein (CP). Analyses were carried out at the Unioeste’s Animal Nutrition Laboratory (LANA/Unioeste).

Urine was collected daily in plastic buckets containing 20 mL of 1:1 HCl to avoid nitrogen volatilization and bacterial proliferation. An aliquot (10% by total volume of urine) was collected daily in polyethylene terephthalate bottles and frozen at −18°C. Subsequently, these samples were homogenized and aliquots taken for the determination of GE. All analyses of feed, urine and feces were performed following the procedures previously reported [12].

### Coefficients of apparent digestibility, values of nutrients and digestible energy, nitrogen and protein balance

The digestibility coefficient of DM, CP, and GE were calculated according to published procedures [13]. The N content of diets, feces and urine samples was analyzed using the Kjeldahl method. The N intake, N excretion (feces and urine), N absorbed, N retention, N retention/N intake, N retention/N absorbed, and total N excretion were then determined.

Quantities of crude protein consumed, crude protein excreted in feces and excreted in urine were obtained by multiplying protein concentrations by the amount of consumed feed, or feces and urine excreted, respectively. From these values, the retained CP, net protein utilization and the biological value of dietary protein, were determined according to a previous study [14].

### Statistical analysis

Before evaluating the results using analysis of variance (ANOVA), the standardized residues analysis of Student (RStudent) was performed to identify outliers. The criterion adopted for identification of outliers was based on a normal distribution curve; values of RStudent greater than or equal to three standard deviations were considered as influential. The normality of the experimental errors and the homogeneity of variances between the treatments for the several variables were previously evaluated using the Shapiro-Wilk and Levine tests, respectively. All parameters had normal distribution and homogeneity.

The statistical model used for the digestibility coefficients and digestible nutrients of the feed was Y_ij_ = m+T_i_+b_j_+ɛ_ij_, wherein: Y_ij_ = observation of the dependent variable in each plot, measured in the i-th protein source, at the j-th block and in k-th replication; m = effect of the overall mean; T_i_ = effect of classes of protein sources, for i = (1, 2, and 3); b_j_ = block effect, for j = (1 and 2); ɛ_ij_ = random errors of the plots associated with level i, to the block j and replication k, independents, homoscedastic and with normal distribution.

The effects of the treatment classes about the dependent variables were verified using ANOVA. The comparisons between treatment averages were performed with Tukey’s test, with a 5% level of significance in all statistical analyses, using SAS software (SAS Inst. Inc. Cary, NC, USA).

For the performance characteristics, the statistical model used was Y_ijk_ = m+P_i_+S_j_+PS_ij_+β (X_ijk_ − X̄…)+ɛ_ijk_. For plasma concentrations of urea and glucose, the statistical model did not include the covariate effect. The effects of the factors included in the model are represented by:

Y_ijk_ = average observation of the dependent variable in each plot, measured in the i-th protein source, in the j-th sex class and in the k-th replication; m = effect of the overall mean; P_i_ = effect of class of protein source, for i = (1, 2, and 3); S_j_ = effect of sex classes, for j = (1 and 2); PS_ij_ = effect of interaction between the i-th class of protein source and the j-th sex class; β = regression coefficient of Y about X; X_ijk_ = average observation of the covariate (initial BW) in each plot, measured in i-th protein class, in j-th sex class and in k-th replicate; X̄… = overall average for the covariate X; ɛ_ijk_ = random error of the plot associated with level i, class j, and replication k.

The effects of protein source, sex and interaction between classes of protein source and sex on the dependent variables were determined using analysis of covariance for the performance characteristics. Comparisons between averages of minimum squares (lsmeans), related to the effect of sex, protein source and blood parameters were performed using Tukey’s test at the 5% probability level. Statistical analyses were performed using the “Proc Univariate” and “General Linear Models” procedures of the SAS software (SAS Inst. Inc. Cary, NC, USA). All data are presented as means with pooled standard error of the mean.

### Growth performance

The animals received feed and water *ad libitum* throughout the experimental period. All the piglets received a pre-weaning feed to reduce weaning stress. Leftover feed was collected, weighed and discounted from feed supplied to calculate average daily feed intake (ADFI, kg/d). The combined weight of the animals recorded on an electronic scale (Rinnert brand, capacity of 1,000 kg) was registered at the beginning and end of each experimental phase. Weights were: 6.88 to 10.02 kg for pre-starter I (27 to 40 days of age), 10.03 to 13.08 kg for pre-starter II (41 to 50 days of age) and 13.09 to 21.28 kg for starter (51 to 65 days of age). Based on these data, average values were determined for ADFI, daily weight gain (DWG, kg/d), and gain to feed ratio (G:F).

### Blood parameters

Urea and glucose were determined with an automated biochemical analyzer (model Flexor EL 200; ELITech Latin America, Brazil), using specific ELITech kits (Clinical Systems, Brazil).

## RESULTS AND DISCUSSION

### Coefficients of apparent digestibility, values of nutrients and digestible energy

No differences were observed between the treatments for the apparent digestibility coefficients, and digestible nutrients (DN) of DM, OM, and DE. However, there was difference (p<0.05) between the treatments for the digestible protein (Table 3).

Bertol and Ludke [15] reported that the type and processing intensity involved in production of SBM may lead to great differences in digestibility coefficients. In the same study, the authors reported apparent dry matter digestibility coefficient (ADMDC) of 86.58% and digestible energy (DE) of 3,515 kcal/kg with diets containing 70% reference diet and 30% SPC. For diets with 70% of the reference diet and 30% SBM, of which the CP values were 44.5% and 46.5%, these results were 87.09% and 87.70% for ADMDC, 3,387 and 3,443 kcal/kg DE, respectively.

The soybean by-product extrusion process improves N digestibility, reduces the concentration of trypsin inhibitors and decreases the antigenic properties of these by-products, resulting in better digestibility and reduced potential for immune reaction to soybean proteins [16]. It is noteworthy that, with increasing age, there is an increase in the digestibility coefficient of some nutrients, including protein and ether extract, which is more accentuated in less digestible diets [17].

Although the values of digestibility coefficient of CP (DCCP) varied from 86.65% to 88.17%, differences were not obtained between the protein sources. The DCCP of SPC was practically analogous to 87.57% obtained by Bertol and Ludke [15]. In addition, the authors reported an DCCP value of 85.91% in one experiment (reference diet - 70% and 30% of SBM with 44.5% of PB), which was slightly lower than that found in this study (86.12%). Studies of the digestibility of FM in pigs are scarce.

The suitable processing of soybean products increases protein breakdown, with greater activity of digestive enzymes, while super processing decreases nutritional value. On the other hand, the lack of heating during processing is also harmful, because the soybean contains anti-nutritional factors interfere with the digestive process [18]. By conjecture, thermal processing of SPC may not have been enough to deactivate the anti-nutritional factors, impairing the availability of amino acids and contributing to the lower digestible protein value in relation to the other experimental diets (Table 3).

The variation observed for DN in relation to DM (%) for the diet containing FM was 2.3 percentage units less than the study of Sulabo et al [6], in which the value was 85.93%. Corroborating these values, Rojas and Stein [19] found greater values in their investigation (88.53%). The digestible DM (%) for the other treatments were discrepant to those determined by Bertol and Ludke [15], for SPC with 62.44% CP (90.84%) and SBM (88.01% and 89.58%), 44.5% and 46.5% CP, respectively.

Oliveira and Stein [4] reported that the increase and improvement in DE concentrations and metabolizable energy (ME) in barrows (BW of 13.94±1.34 kg) fed with SPC compared to those receiving a diet containing SBM was due to the process used to remove carbohydrates, such as oligosaccharides. These results are linked to the fact that pigs do not secrete β-galactosidase, an enzyme necessary for the hydrolysis of raffinose and stachyose, therefore these are not enzymatically digested in the small intestine. The removal of oligosaccharides from SPC resulted in greater concentrations of CP and greater concentrations of DE.

Rojas and Stein [19] determined the concentrations of DE and ME in pigs fed with fermented soybean meal (FSM), conventional soybean meal (CSM) and FM and obtained values consistent with those in this study for DE of FM (3,827 kcal/kg), but not for CSM (4,553 kcal/kg). The authors considered that this may be a consequence of a greater percentage of ash in the FM used. In addition, the concentration of CP and DE in FM compared with FSM and CSM is most likely due to amino acid composition. However, the results indicate that the amino acids and GE of FM are poorly digested, and consequently the DE and ME were lower in FM than in FSM. This finding is similar with the result of this study when compared to protein sources of vegetable origin.

### Nitrogen and protein balance

With respect to the nitrogen and protein balance in piglets, the difference for variable total nitrogen excretion (TNE, g/kg BW^0.75^/d) was significant (p<0.05). There was no effect of treatment on the other variables (Table 4).

Dourmad et al [20] reported that approximately 66.66% of ingested N is excreted in pigs. Thus, the increase in use of protein compounds decreases urea synthesis, and coincidentally N retention must be maximized in animals fed amino acid balanced feeds. It is possible that the control diet promoted a greater catabolism of the amino acid molecules, consequently the amount of N in feces and urine was numerically greater, which caused a difference in TNE (g/kg BW^0.75^/d), since both variables are used to determine absorbed and retained N.

Bertechini [21] claims that N excretion commonly occurs at a basal ration, being influenced by two situations: process of gluconeogenesis (fasting metabolism) and when there is excessive intake of protein. Finally, it is acceptable that ingested N was not influenced significantly by the treatments, from the results of N intake (27.09, 26.44, and 28.57 g/d; Table 4) which supports the author’s explanation about excretion of N.

According to Dourmad et al [20], the reduction in urinary N elimination results from the decrease in the amount of N absorbed through of the intestine and the increase in the use of N. The lack of effect of protein sources on the crude protein intake can be correlated to the absence of significance for the variable ingested N, considering that both are interconnected [22]. The net protein utilization of 64.62% to 67.22% in the current study were similar to 65.5% and 60.8% obtained by Oliveira et al [23], who worked with growing pigs and concentrations of 12% and 14% of CP in feeds.

### Growth performance

All the piglets remained healthy throughout the duration of the experiment period. There was a significant effect of interaction (p<0.05) between sex and protein sources on the ADFI, DWG, and G/F in pre-starter I phase, ADFI in pre-starter II phase, FBW in starter phase and ADFI, DWG, and FBW for the total period. In addition, in pre-starter I and starter phases, and total period, there was a difference (p<0.05) in ADFI between the sexes (Table 5). A significant difference (p<0.05) was obtained between the protein sources in starter phase and total period for ADFI, DWG, and FBW (Table 5).

For the effect of sex within diet, the females presented the highest values in relation to males for all the variables of growth performance during the experiment. In contrast, the effect of diet within sex, there was only difference between males, with male piglets fed with SPC+SBM showing the best results (Table 5).

These data are not consistent with results of other studies because differences in performance result from changes that accompany the sexual development of the animal, which cannot be examined at an early age. Analogous effects of sex on performance were reported by Silva et al [24], in a study of sweetener in drinking water, in which females showed better DWG and ADFI in phases between 30 to 45 d and 45 to 62 d. The authors argued an assumption that males would overcome the weight gain of the females by the age of slaughter.

These results are pertinent to those obtained in this study (Table 5), where SBM presents some disadvantages for feeding piglets at early age, related to low digestibility and the presence of antigenic proteins, glycinin and β-conglycinin. These anti-nutritional properties cause changes in intestinal mucosa, and, consequently, in growth rate of piglets after weaning [3] by reducing the absorptive and digestive capacity of the gastrointestinal tract.

Similarly, Bertol et al [3] tested soybean by-products on the SPC for piglets weaned at 21 days of age and reported that the partial replacement of SBM by any soybean processed sources improved the growth performance.

It is possible that the protein quality of the studied sources caused differences in final weigh, due the physiological and biochemical changes of functions of the gastrointestinal tract, causing a reduction in growth performance because of the degradation of tissue protein. Bertechini [21] argues that the composition of protein sources can lead to amino acid imbalance that does not meet metabolic requirements. As a result, the intake of an unbalanced diet compromises at the decrease of consumption and growth retardation.

### Blood parameters

There was no interaction between blood parameters, urea and glucose and protein sources and sex in pre-starter II and starter phases. However, for both variables in the pre-starter II phase there was a difference (p<0.05) between the protein sources (Table 6). The values found are close to the established range of 13 to 45 mg/dL for urea and 65 to 106 mg/dL for glucose according to the specific ELITech kits (ELITech Group, Paris, [Commune Puteaux], France). A difference (p<0.05) was also obtained between the protein sources and sex in the urea variable during the starter phase (Table 6).

In this context, it is possible that the protein quality of FM and SPC is relatively greater compared with to the control diet (SBM), considering the plasma urea concentration (PUC). It also suggests that the low biological value of the protein is closely related to the catabolism of amino acids [25].

In the growth phase of animals, the nutritional and physiological needs change, as do protein concentrations in feed, until a plateau of protein requirement is established. Notably, in starter phase the pigs showed a significant increase in blood urea concentration in relation to the previous phase. It is possibly due to the composition of the feed in pre-starter II phase, with ingredients that present better quality and digestibility of the CP generating smaller concentrations of metabolites.

The increase in the activity of enzymes involved in the urea cycle is essential for conversion of ammonia, generated from the oxidative deamination of protein metabolites, to urea. Metabolic pathways for urea synthesis by the liver use energy [26], in which the most commonly used fuel is glucose, a high energy monosaccharide which breaks down inside cells to release stored energy as molecules of adenosine triphosphate. The increase in urea or PUC synthesis could increase the energy expenditure by the liver and concomitantly reduce the glucose that would be destined for other purposes.

It is recognized that sex affects protein metabolism, especially in the starter phase, where CP concentrations are partially discrepant [9]. Therefore, the increase of the PUC could be caused by increasing protein concentrations in the diet provided to the piglets.

The rate of muscular deposition in entire males was greater and more accelerated in relation to females, with maturity of protein deposition later, but in greater amount of use of nitrogen compounds (urea) (Table 6).

## CONCLUSION

The use of SBM-based feeds as a conventional protein source and SPC+SBM for pigs in the starter phase affects total excretion of nitrogen and reduces the digestible protein, respectively. The use of SPC+SBM in piglet feed improves growth performance parameters in the starter phase and in the total period and reduces PUCs in the pre-starter II phase compared to the use of SBM-based feeds only. The use of FM+SBM-based feeds for piglets in the pre-starter II phase reduced plasma glucose concentrations compared to the use of SPC+SBM and SBM-based feeds only.

## IMPLICATIONS

Processed soybean products have low trypsin inhibitors, lower concentrations of antigenic proteins and lectin contents. A factor to be considered in the quality of these products is inadequate processing of the raw material, causing a low nutritional value and lower coefficient of digestibility of the ingredients. Fish meal is a protein of high biological value, but its quality depends of the type and species of fish, freshness of the fish before processing and the method used for processing; these factors may result in products with low protein content and excess minerals, which reduce its nutritional value.

## Figures and Tables

**Table 1:** Centesimal and chemical composition of experimental diets for piglets in nursery phase (as-fed basis)

Items	Protein sources											

	SBM+FM				SBM+SPC				SBM			

	Experimental phases1) (6.00 to 22.00 kg)											

	PW	PI	PII	S	PW	PI	PII	S	PW	PI	PII	S
Corn	38.0	49.2	55.2	59.8	38.0	49.2	55.2	59.8	38.4	47.8	54.0	59.3
Soybean meal 45.22%	9.0	18.0	25.0	30.0	9.0	18.0	25.0	30.0	12.6	22.9	29.2	32.0
Soybean oil	4.0	3.4	2.8	2.7	4.0	3.4	2.8	2.7	4.0	3.4	2.8	2.7
Fish meal	4.0	3.5	3.0	1.5	-	-	-	-	-	-	-	-
SPC	-	-	-	-	4.0	3.5	3.0	1.5	-	-	-	-
Nucleus2)	45.0	26.0	14.0	6.0	45.0	26.0	14.0	6.0	45.0	26.0	14.0	6.0
Total (%)	100.0	100.0	100.0	100.0	100.0	100.0	100.0	100.0	100.0	100.0	100.0	100.0
Calculated composition												
Crude protein (%)	18.2	18.5	19.1	20.1	18.2	18.5	19.1	20.0	18.2	18.4	19.0	20.0
Lactose (%)	14.7	7.1	2.6	-	14.7	7.1	2.6	-	14.7	7.1	2.6	-
ME (kcal/kg)	3,726	3,555	3,427	3,401	3,708	3,556	3,416	3,400	3,329	3,501	3,506	3,497
Lysine (%)	1.417	1.372	1.345	1.321	1.416	1.373	1.346	1.320	1.416	1.374	1.347	1.320
Methionine (%)	0.52	0.52	0.52	0.48	0.51	0.51	0.51	0.48	0.52	0.52	0.52	0.48
Calcium (%)	0.48	0.55	0.70	0.72	0.39	0.38	0.70	0.61	0.39	0.38	0.70	0.70
STTD phosphorus (%)	0.45	0.39	0.35	0.34	0.43	0.37	0.34	0.33	0.44	0.38	0.34	0.33
Sodium (%)	0.33	0.28	0.23	0.24	0.30	0.25	0.20	0.22	0.30	0.25	0.20	0.22

SBM, soybean meal; FM, fish meal; SPC, soybean protein concentrate; ME, metabolizable energy; STTD, standardized total tract digestible.

1) PW, post-weaning; PI and PII, pre-starter I and II; S, starter.

2) Composition per feed and phase: SBM+FM (%): Mg monoxide 0.12, 0.15, 0.18, 0.19; Cl sulfate 0.67, 0.52, 0.42, 0.40; K iodate 1.05, 0.89, 0.80, 0.79 (mg/kg); Cu sulfate 4.5, 4.5, 154.5, 154.5; Organic Cu 117, 117, 4.5, 4.5; Fe sulfate 70; I 1.2; Mn sulfate 30.2; Org. Mn 10.1; Niacin 35.1; Sodium selenite 0.30; Zn oxide 2,500; Org. Zn 10.0; Vitamin K_3_ 4.0; Vitamin B_1_ 2.1; Vitamin B_2_ 10.2; Vitamin B_6_ 6.0; Vitamin B_12_ 0.04; Folic acid 1.01; Pantothenic acid 19.9; Biotin 0.15; Choline chloride 1,347, 1,445, 1,421, 1,479; BHT 3.76; Ethoxyquin 15.0 (IU/kg); Vitamin A 14,100; Vitamin D_3_ 2,820; Vitamin E 80.3; SBM+SPC (%): Cl sulfate 0.62, 0.48, 0.38, 0.39; K iodate 1.10, 0.94, 0.84, 0.80 (mg/kg): Choline chloride 1,245, 1,356, 1,343, 1,432; SBM (%): Mg monoxide 0.13, 0.15, 0.18, 0.19; Cl sulfate 0.62, 0.48, 0.38, 0.39; K iodate 1.11, 0.94, 0.84, 0.81 (mg/kg): Choline chloride 1,281, 1,388, 1,370, 1,447. Values represent the amount of mineral or vitamin per phase PW, PI, PII; S. Only the amount of chlorine sulfate, potassium iodate and choline chloride are different between the feeds, and magnesium monoxide for the SBM feed.

**Table 2:** Nucleus composition (g/kg) of experimental feeds for piglets in nursery phase (as-fed basis)

Concentrations of warranty (g/kg)1)	Post-weaning2)	Pre-starter I2)	Pre-starter II2)	Starter2)
Crude protein (Min)	186.00	280.00	260.00	270.00
Ether extract (Min)	55.00	13.00	17.00	26.00
Mineral matter (Max)	50.00	150.00	185.00	500.00
Calcium (Min to Max)	3.00 to 5.00	1.30 to 1.50	20.0 to 40.0	100.00 to 140.00
Phosphorus (Min)	4.50	10.00	16.00	35.00
Sodium (Min)	-	-	-	35.00
Lysine (Min)	15.00	32.00	40.00	55.00
Methionine (Min)	5.85	15.00	22.00	37.00
Threonine (Min)	-	-	-	28.00
Crude fiber (Max)	14.00	7.00	5.00	8.00
Phytase unit/kg (Min)	500.00	-	-	8.30

1) Minimum and maximum values.

2) Basic composition of the nucleus, post-weaning: vegetable fatty acid (ACFA), acidifying additive, aromatizing additive, enzymatic additive, bentonite, biotin, calcium carbonate, choline chloride, sodium chloride, grape seed extract (polyphenols), phytase, dicalcium phosphate, soybean grain extruded, potassium iodate, L-lysine, DL-methionine, L-threonine, L-tryptophan, degummed soybean oil, zinc oxide, palatabilizant, calcium pantotenate, blood plasma, sodium selenite, whey powder, milk-sugar, cobalt sulfate, copper sulfate, iron sulfate, manganese sulphate, colistin sulfate; pre-starter I and II: same composition of PW, except for aromatizing additive, bentonite, grape seed extract, degummed soybean oil and milk-sugar; starter: same composition of Pre-starter I and II, except for enzymatic additive, soybean grain extruded, L-tryptophan, blood plasma and whey powder.

**Table 3:** Coefficients of apparent digestibility, values of nutrients and digestible energy of piglets in starter phase in Exp 1

Item	Treatments1)			SEM	p-value2)

	FM	SPC	SBM		
Coefficients of apparent digestibility					
Dry matter (%)	85.9	87.4	85.4	0.46	0.185
Crude protein (%)	86.9	88.1	86.6	0.50	0.490
Organic matter (%)	88.2	89.7	87.9	0.40	0.175
Gross energy (%)	87.3	88.6	87.3	0.40	0.310
Values of nutrients and digestible energy					
Dry matter (%)	83.6	84.8	82.6	0.45	0.169
Digestible protein (%)	17.8^a^	16.7^b^	18.1^a^	0.16	<0.001
Organic matter (%)	81.6	82.5	80.8	0.37	0.167
Digestible energy (kcal/kg)	3,810	3,842	3,851	17.05	0.606

SEM, pooled standard error of the means.

1) FM, fish meal; SPC, soybean protein concentrate; SBM, soybean meal (n = 8).

2) Protein sources (diets) effect.

a,b Values followed by different lowercase letters, in row, differ according to Tukey’s test, at 5% probability level.

**Table 4:** Nitrogen (N) and protein balance in entire male piglets in starter phase fed with different protein sources in Exp. 1

Item1)	Treatments2)			SEM	p-value3)

	FM	SPC	SBM		
N intake (g/d)	27.09	26.44	28.57	1.38	0.772
N intake (g/kg BW^0.75^/d)	2.41	2.30	2.52	0.07	0.453
N fecal (g/d)	3.51	3.20	3.98	0.27	0.526
N fecal (g/kg BW^0.75^/d)	0.31	0.27	0.34	0.01	0.333
N urine (g/d)	5.39	5.42	5.98	0.30	0.610
N urine (g/kg BW^0.75^/d)	0.48	0.47	0.54	0.02	0.242
N absorbed (g/d)	23.57	23.23	24.14	1.14	0.894
N absorbed (g/kg BW^0.75^/d)	2.09	2.03	2.16	0.05	0.582
N retained (g/d)	18.17	17.80	18.16	0.96	0.961
N retained (g/kg BW^0.75^/d)	1.61	1.55	1.61	0.05	0.886
N retained/intake (%)	66.80	67.22	64.62	0.79	0.968
N retained/absorbed (%)	76.80	76.30	74.72	0.99	0.692
Total N excretion (g/d)	8.91	8.63	10.02	0.49	0.468
Total N excretion (g/kg BW^0.75^/d)	0.79^b^	0.75^b^	0.88^a^	0.02	0.049

SEM, pooled standard error of the means; BW, body weight.

1) P = 6.25×N.

2) FM, fish meal; SPC, soybean protein concentrate; SBM, soybean meal (n = 8).

3) Protein sources (diets) effect.

a,b Values followed by different lowercase letters, in row, differ according to Tukey’s test, at 5% probability level.

**Table 5:** Growth performance of piglets fed with different protein sources in nursery phase in Exp 2

Item	Protein sources1)			Sex		SEM	p-value2)		
		
	FM	SPC	SBM	Male	Female		Diets	Sex	Diets×sex
Pre-starter I phase (6.88 to 10.02 kg)									
ADFI	0.27	0.28	0.27	0.27^b^	0.28^a^	0.003	0.478	0.035	0.015
DWG	0.21	0.22	0.21	0.21	0.21	0.005	0.510	0.534	0.004
G:F	0.76	0.79	0.77	0.78	0.77	0.01	0.741	0.685	0.042
FBW	10.31	10.19	9.59	9.98	10.07	0.15	0.468	0.335	0.282
Pre-starter II phase (10.03 to 13.08 kg)									
ADFI	0.48	0.51	0.46	0.47	0.49	0.01	0.168	0.197	0.017
DWG	0.31	0.33	0.30	0.31	0.32	0.008	0.408	0.502	0.199
G:F	0.66	0.66	0.65	0.66	0.65	0.01	0.963	0.668	0.391
FBW	13.36	13.33	12.57	13.05	13.13	0.17	0.447	0.526	0.093
Starter phase (13.09 to 21.28 kg)									
ADFI	0.79^ab^	0.80^a^	0.71^b^	0.73^b^	0.80^a^	0.01	0.017	<0.001	0.196
DWG	0.53^ab^	0.56^a^	0.48^b^	0.51	0.54	0.01	0.021	0.071	0.101
G:F	0.67	0.70	0.68	0.69	0.67	0.009	0.686	0.265	0.623
FBW	21.62^ab^	22.18^a^	20.09^b^	21.01	21.57	0.26	0.001	0.091	0.022
Total period (6.88 to 21.28 kg)									
ADFI	0.47^ab^	0.49^a^	0.44^b^	0.45^b^	0.48^a^	0.007	0.022	0.002	0.045
DWG	0.33^ab^	0.35^a^	0.31^b^	0.32	0.34	0.005	0.004	0.060	0.011
G:F	0.71	0.72	0.71	0.72	0.70	0.005	0.398	0.134	0.372
FBW	21.62^ab^	22.18^a^	20.09^b^	21.01	21.57	0.26	0.001	0.091	0.022

SEM, pooled standard error of the means; ADFI, average daily feed intake; DWG, daily weight gain; G:F, gain to feed ratio; FBW, final body weight.

1) FM, fish meal; SPC, soybean protein concentrate; SBM, soybean meal.

2) Protein sources (diets) effect; Sex effect; Diets×sex, interaction between diets and sex.

a,b Lsmeans followed by different lowercase letters, in row, differ according to Tukey’s test, at 5% probability level.

**Table 6:** Plasma urea and glucose (mg/dL) in pre-starter II and starter phases according with combinations of protein sources and sex in Exp 2

Item	Protein sources1)			Sex		SEM	p-value2)		
		
	FM	SPC	SBM	Female	Male		Diets	Sex	Diets×sex
Pre-starter II phase (10.03 to 13.08 kg)									
Urea	34.02^b^	33.88^b^	38.80^a^	36.93	34.19	0.82	0.024	0.101	0.560
Glucose	103.76^b^	110.85^a^	109.72^a^	106.64	109.58	0.87	0.001	0.089	0.957
Starter phase (13.09 to 21.28 kg)									
Urea	50.33^b^	53.00^ab^	58.23^a^	57.11^a^	50.60^b^	1.09	0.010	0.003	0.408
Glucose	100.4	99.80	100.66	98.81	101.76	1.13	0.949	0.218	0.063

SEM, pooled standard error of the means.

1) FM, fish meal; SPC, soybean protein concentrate; SBM, soybean meal.

2) Protein sources (diets) effect; Sex effect; Diets×sex, interaction between diets and sex.

a,b Lsmeans followed by different lowercase letters, in row, differ according to Tukey’s test, at 5% probability level.
